# Increased internalization of complement inhibitor CD59 may contribute to endothelial inflammation in obstructive sleep apnea

**DOI:** 10.1126/scitranslmed.aad0634

**Published:** 2016-01-06

**Authors:** Memet Emin, Gang Wang, Francesco Castagna, Josanna Rodriguez-Lopez, Romina Wahab, Jing Wang, Tessa Adams, Ying Wei, Sanja Jelic

**Affiliations:** 1 Division of Pulmonary, Allergy, and Critical Care Medicine, Columbia University College of Physicians and Surgeons, New York, NY 10032, USA; 2 Division of Biostatistics, Columbia University College of Physicians and Surgeons, New York, NY 10032, USA

## Abstract

Obstructive sleep apnea (OSA), characterized by intermittent hypoxia (IH) during transient cessation of breathing, triples the risk for cardiovascular diseases. We used a phage display peptide library as an unbiased approach to investigate whether IH, which is specific to OSA, activates endothelial cells (ECs) in a distinctive manner. The target of a differentially bound peptide on ECs collected from OSA patients was identified as CD59, a major complement inhibitor that protects ECs from the membrane attack complex (MAC). A decreased proportion of CD59 is located on the EC surface in OSA patients compared with controls, suggesting reduced protection against complement attack. In vitro, IH promoted endothelial inflammation predominantly via augmented internalization of CD59 and consequent MAC deposition. Increased internalization of endothelial CD59 in IH appeared to be cholesterol-dependent and was reversed by statins in a CD59-dependent manner. These studies suggest that reduced complement inhibition may mediate endothelial inflammation and increase vascular risk in OSA patients.

## Introduction

Obstructive sleep apnea (OSA), a condition that affects a quarter of Western adults, triples the risk for hypertension, ischemic stroke, coronary artery disease, and venous thromboembolism and increases all-cause mortality (1–7). OSA is characterized by repetitive cessation of breathing, resulting in episodes of hypoxia/reoxygenation that activate the endothelium, a key step in the development and progression of cardiovascular diseases (8–10). Consequent platelet aggregation, leukocyte and monocyte adhesion, and macrophage activation likely contribute to cardiovascular risk (9, 11, 12). However, the initial stimulus that mediates endothelial activation in OSA has not been characterized.

The cyclic nature of the hypoxic insult clearly distinguishes OSA from other conditions that activate the endothelium, such as hypertension, diabetes, or elevated low-density lipoprotein (LDL) cholesterol. Furthermore, diseases associated with continuous hypoxia, such as chronic obstructive pulmonary disease, interstitial lung disease, and pulmonary hypertension, are not considered independent risk factors for cardiovascular disease after adjustment for common confounders such as smoking (13). In contrast, repetitive hypoxia/reoxygenation, a phenomenon specific to OSA that is usually associated with relatively mild hypoxemia, clearly promotes vascular inflammation and accelerates atherosclerosis, whereas treatment of OSA with continuous positive airway pressure (CPAP) promotes regression of atherosclerosis (10, 14–16). This led us to hypothesize that repetitive hypoxia/reoxygenation that characterizes OSA activates endothelium in a distinctive manner.

To gain insight into pathways that mediate vascular risk in OSA, we used an unbiased approach using a phage display library and mass spectrometry to identify differentially expressed plasma membrane proteins on endothelial cells (ECs) harvested from patients with OSA and OSA-free controls. This resulted in the discovery that OSA patients have altered cellular distribution of CD59, with a greater proportion found intracellularly in OSA patients compared with controls. CD59, a major complement regulator, is a glycosylphosphatidylinositol (GPI)–anchored plasma membrane protein that inhibits the formation of the terminal complement membrane attack complex (MAC) and protects host cells from complement-mediated injury (17). Our studies showed that intermittent hypoxia (IH) decreases protection against complement activity by promoting the internalization of CD59 and thus deposition of the MAC on ECs, suggesting a mechanism of vascular injury in OSA. Increased internalization of CD59 appeared to be dependent on cholesterol-enriched lipid raft formation in ECs and was blocked by statins, suggesting an additional protective effect of statins in OSA patients.

## Results

### Binding of phage display peptide to complement regulator CD59 is increased in ECs of OSA patients

**Fig. 1:**
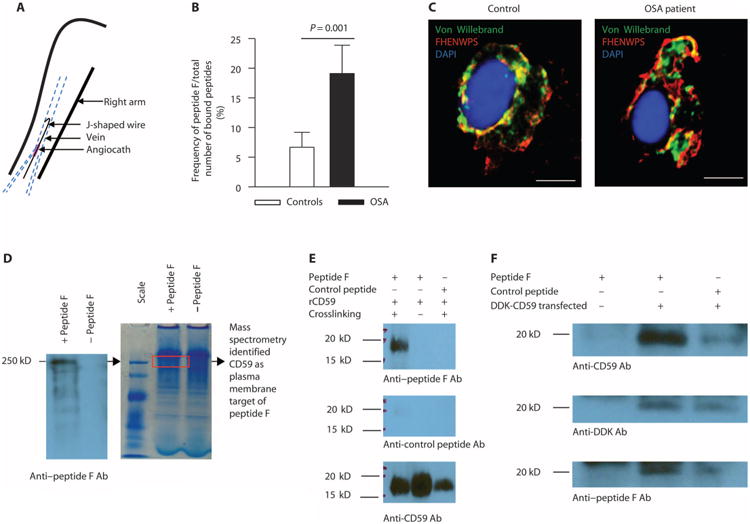
EC plasma membrane proteins are differentially expressed in OSA patients and OSA-free controls (**A**) Harvesting of venous ECs from study participants (*n* = 76 OSA patients and *n* = 52 controls, demographics in table S1). (**B**) Frequency of peptide F binding to ECs harvested from OSA patients [*n* = 18: 45% male; age, 44.6 ± 2.4 years; body mass index (BMI), 38.1 ± 2.1 kg/m^2^; apnea-hypopnea index (AHI), 26.2 ± 6.2 events/hour; oxygen desaturation index (ODI), 11.4 ± 2.8 events/hour] and controls (*n* = 20: 55% male; age, 40.9 ± 2.7 years; BMI, 27.9 ± 1.3 kg/m^2^; AHI, 1.3 ± 0.4; ODI, 0.37 ± 0.3 events/hour) expressed as a percentage of the total number of bound peptides (mean±SE; two-sided Student's *t* test adjusted for age, gender, and BMI shown in fig. S2, *P* = 0.001). (**C**) Representative confocal image of peptide F binding to ECs harvested from OSA patients (*n* = 10: 30% male; age, 50.3 ± 3.2 years; BMI, 34.9 ± 1.8 kg/m^2^; AHI, 19.4 ± 5.8 events/hour; ODI, 12.3 ± 3.3 events/hour) and controls (*n* = 10: 60% male; age, 38.4 ± 3.4 years; BMI, 29.4 ± 1.6 kg/m^2^; AHI, 1.9 ± 0.7 events/hour; ODI, 1.1 ± 0.6 events/hour); ECs are identified by immunofluorescence for von Willebrand factor. Scale bars, 10 μm. (**D**) Native polyacrylamide gel electrophoresis (PAGE) and Western blotting with anti–peptide F antibodies (Ab) of human umbilical vein endothelial cell (HUVEC) lysate incubated with or without peptide F. The region of bound peptide F (a single major band at molecular weight of 250 kD) was cut out from the gel (red rectangle), subjected to trypsin digestion, and analyzed by mass spectrometry. Among proteins identified in this region, CD59 was the only one that is expressed on the plasma membrane. (**E**) In vitro binding of re-combinant CD59 (rCD59; molecular weight, 18 to 20 kD) to peptide F (molecular weight, 700 to 800 daltons) after cross-linking but not to control peptide (Western blots probed with anti–peptide F, anti–control peptide, and anti-CD59 antibodies; *n* = 3). (**F**) Immunoprecipitation with beads coated with anti-DDK antibodies of human embryonic kidney (HEK) 293 cells transfected with Myc-DDK–tagged human CD59 plasmid incubated with peptide F, followed by SDS-PAGE and Western blotting with anti-CD59, anti-DDK, and anti–peptide F antibodies, showing specific binding of peptide F to CD59 (*n* = 3).

We hypothesized that repetitive hypoxia/reoxygenation during transient cessation of breathing, which resembles recurrent ischemia-reperfusion injury and is specific to OSA, may alter EC receptors in a manner that differs from endothelial activation in other cardiovascular diseases. We used a phage display library to profile ECs harvested from OSA patients and OSA-free controls (18). ECs were scraped from the forearm vein with a J-shaped endovascular wire threaded through an angiocath (Fig. 1A). Although OSA patients (*n* = 76) were slightly older than controls (*n* = 52), the two groups were matched for gender, adiposity, daytime sleepiness, blood pressure, and comorbidities (table S1). Scraped ECs were purified using magnetic beads coated with endothelial-specific antibodies (CD146) and exposed to a library of phage displaying a large variety of seven–amino acid peptides. Among peptides that bound to scraped ECs, peptide FHENWPS (peptide F) showed the greatest enrichment for binding to ECs from OSA patients compared with controls (Fig. 1B). We did not detect binding of peptide F to bovine serum albumin or polystyrene plates (table S2). Confocal microscopy showed both plasma membrane and intracellular binding of peptide F in scraped ECs, which were identified on slides by positive immunofluorescence for von Willebrand factor (Fig. 1C) (14). This was expected because we had previously observed that scraped ECs are leaky (14). Binding of peptide F to intracellular sites appeared to be particularly prominent in ECs from OSA patients (Fig. 1C).

To identify molecular targets of peptide F, we first carried out SDS-PAGE of HUVEC lysates incubated with peptide F with or without cross-linking and then performed Western blotting with antibody to peptide F. However, no specific signal could be detected, possibly indicating a con-formational target. Thus, we next incubated HUVEC lysate with peptide F, followed by native PAGE and Western blotting with anti–peptide F antibodies (Fig. 1D). This revealed a single major band at molecular weight of 250 kD (Fig. 1D). The region of bound peptide F was cut out from the gel, subjected to trypsin digestion, and analyzed by mass spectrometry. Among proteins identified in this region, CD59 was the only one that is expressed on the plasma membrane and was thus selected for further study. Although the molecular weight of CD59 is 18 to 20 kD, its migration at higher molecular weight in the native gel likely reflects its presence in membrane fragments and/or in higher-order complexes.

A direct interaction between CD59 and peptide F was first confirmed by the in vitro binding of recombinant CD59 (molecular weight, 18 to 20 kD) to peptide F (molecular weight, 700 to 800 daltons) after cross-linking but not to a control peptide (Western blot probed with anti–peptide F antibodies; Fig. 1E). To further demonstrate interaction between CD59 and peptide F, we transfected HEK293 cells with Myc-DDK–tagged human CD59 plasmid. After incubation and cross-linking of transfected cells with peptide F, immunoprecipitation with beads coated with anti-DDK antibodies was performed, followed by SDS-PAGE and Western blotting with anti-CD59, anti-DDK, and anti–peptide F antibodies. This confirmed specific binding of peptide F to CD59 (Fig. 1F).

### Cellular distribution of CD59 is altered in OSA

**Fig. 2:**
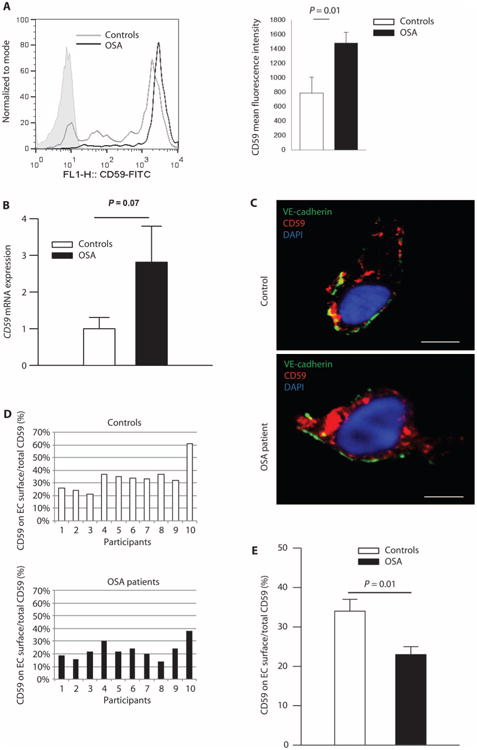
Cellular distribution of CD59 is altered in OSA (**A**) Mean fluorescence intensity of total cellular CD59 (flow cytometry, shaded area represents isotype control; the quantity of cells on the *y* axis is expressed as percentage of total specimens). Bar graph quantitates total CD59 fluorescence (*n* = 8 OSA patients: 62% male; age, 42.3 ± 5.2 years; BMI, 35.9 ± 2.0 kg/m^2^; AHI, 21.3 ± 8.7 events/hour; ODI, 12.9 ± 4.5 events/hour; *n* = 9 controls: 33% male; age, 36.7 ± 4.6 years; BMI, 35.8 ± 3.2 kg/m^2^; AHI, 3.2 ± 0.7 events/hour; ODI, 2.0 ± 0.5 events/hour) (mean ± SE; two-sided exact permutation test). (**B**) Quantitation of the *CD59* mRNA expression in ECs harvested from OSA patients (*n* = 21: 67% male; age, 45.5 ± 2.7 years; BMI, 35.6 ± 2.1 kg/m^2^; AHI, 23.5 ± 5.5 events/hour; ODI, 19.3 ± 6.1 events/hour) expressed as a fold change over controls (*n* = 15: 27% male; age, 34.1 ± 3.4 years; BMI, 36.6 ± 3.0 kg/m^2^; AHI, 1.3 ± 0.4 events/hour; ODI, 0.3 ± 0.2 events/hour) (two-sided Student's *t* test adjusted for age, gender, and BMI, shown in fig. S2). (**C**) Representative confocal images of cellular distribution of CD59 in ECs harvested from OSA patients (*n* = 10: 30% male; age, 50.3 ± 3.2 years; BMI, 34.9 ± 1.8 kg/m^2^; AHI, 19.4 ± 5.8 events/hour; ODI, 12.3 ± 3.3 events/hour) and controls (*n* = 10: 40% male; age, 34.5 ± 3.7 years; BMI, 34.1 ± 3.0 kg/m^2^; AHI, 2.2 ± 0.6 events/hour; ODI, 1.4 ± 0.6 events/hour); EC plasma membrane is identified by immunofluorescence for vascular endothelial (VE)–cadherin (CD144). Scale bars, 10 μm. (**D**) Histogram representing the percentage of total endothelial CD59 located on the plasma membrane for individual patients [*n* = 10 OSA patients; *n* = 10 controls; demographics in the legend for (C)]. (**E**) Quantitation of the percentage of total endothelial CD59 located on the plasma membrane [*n* = 10 OSA patients; *n* = 10 controls; demographics in the legend for (C)]. Linear regression confirmed that the difference in plasma membrane expression of CD59 is not confounded by age, gender, or BMI (fig. S2) (mean ± SE; two-sided exact permutation test). FITC, fluorescein isothiocyanate.

On the basis of the identification of CD59 as the target of peptide F, we next used flow cytometry to confirm increased CD59 in ECs from OSA patients. Expression of CD59 in ECs scraped from OSA patients was twofold greater than that from controls (mean fluorescence ± SE, 1479 ± 220 versus 763 ± 146; two-sided Student's *t* test, *P* = 0.01) (Fig. 2A). Because scraped ECs are leaky as a result of the harvesting process, fluorescence intensity on flow cytometry represents both plasma membrane and intracellular expression of CD59. The expression of CD59 was similar in platelets and leukocytes in OSA patients and controls (fig. S1), suggesting that the effects of IH on CD59 localization and function may be limited (or at least the most pronounced) to the vascular endothelium in OSA. Consistent with the overall increase in CD59 protein in OSA ECs, expression of *CD59* mRNA was increased twofold in OSA patients compared with controls, but this did not reach significance (two-sided Student's *t* test, *P* = 0.07) (Fig. 2B). Linear regression confirmed that the difference in total cellular protein and mRNA expression of CD59 is not confounded by age, gender, or BMI (fig. S1). Quantitative confocal microscopy revealed that, in contrast to controls, most of the endothelial CD59 protein in OSA patients is intracellular (Fig. 2C). The proportion of total CD59 present on the EC plasma membrane, which is the site of its protective activity, was reduced in OSA patients (*n* = 10) compared with controls (*n* = 10) (two-sided exact permutation test, *P* = 0.01) (Fig. 2, D and E). These data suggest that although the overall amount of CD59 is increased in ECs from OSA patients, there is a striking difference in its cellular distribution, with a greater proportion of CD59 found intracellularly in OSA patients. This suggested the hypothesis that IH might promote the internalization of CD59, thus limiting its effectiveness in controlling complement activation.

### Endocytosis of endothelial plasma membrane CD59 is increased in IH

**Fig. 3:**
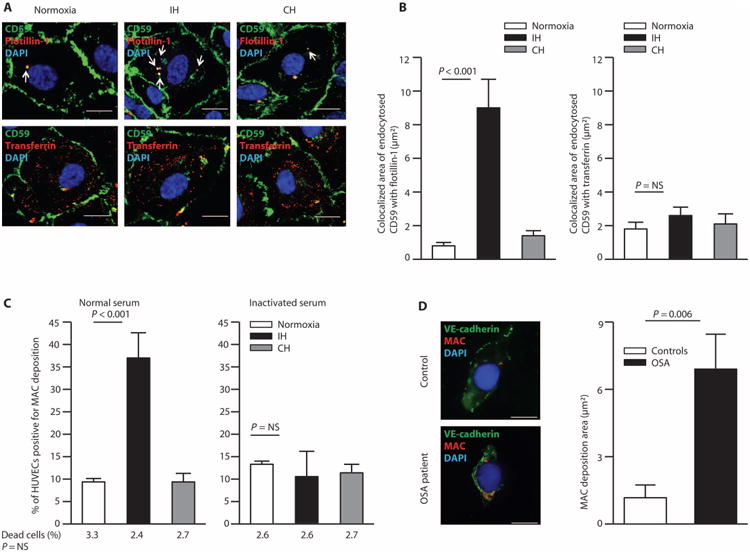
IH increases endocytosis of endothelial CD59 and consequent MAC deposition (**A**) Representative confocal images of CD59 endocytosis in HUVECs in normoxia, IH, and continuous hypoxia (CH). Arrows indicate endocytosed CD59 with flotillin (colocalized area in μm^2^). Endocytosis mediated by transferrin is shown on the bottom panel. Scale bars, 10 μm. (**B**) Quantitation of colocalized area of endocytosed CD59 with flotillin-1 and transferrin (*n* = 8). (**C**) Quantitation of the percentage of HUVECs positive for MAC deposition in normoxia, IH, and continuous hypoxia (flow cytometry), indicating the percentage of dead cells in each condition below the graph (identified by propidium iodide staining) (*n* = 8). (**D**) Representative confocal images of MAC deposition on ECs harvested from OSA patients and controls. Scale bars, 10 μm. Bar graph quantitates MAC deposition area (μm^2^) on ECs of OSA patients (*n* = 8: 62% male; age, 39.0 ± 4.2 years; BMI, 37.2 ± 3.5 kg/m^2^; AHI, 13.7 ± 2.4 events/hour; ODI, 11.5 ± 2.8 events/hour) and controls (*n* = 10: 33% male; age, 39.4 ± 4.4 years; BMI, 34.1 ± 3.4 kg/m^2^; AHI, 1.9 ± 0.9 events/hour; ODI, 1.7 ± 1.0 events/hour). Linear regression confirmed that the difference in MAC deposition on ECs among groups is not confounded by age, gender, or BMI (fig. S2). All data throughout the figure are shown as means ± SE (two-sided exact permutation test). NS, not significant.

To address this hypothesis, we investigated whether endocytosis of endothelial CD59 is affected by IH (alternating periods of 30-min normoxia/30-min hypoxia at 2% oxygen for a total of 8 hours) using cultured HUVECs. Endocytosis of CD59 is clathrin- and dynamin-independent and involves the scaffolding protein flotillin (19–21). To take a snapshot of endocytosis in live cells, HUVECs were incubated with primary antibodies to CD59 and flotillin-1 for 5 min at 37°C to allow endocytosis, then washed, fixed, and permeabilized, and then incubated with secondary antibodies to detect plasma membrane CD59 and internalized CD59 with flotillin (Fig. 3, A and B). Consistent with our hypothesis, endocytosis of CD59 with flotillin was markedly increased in IH; in contrast, CD59 did not colocalize with endocytosed transferrin (Fig. 3, A and B). In contrast, this was not observed with continuous hypoxia (2% oxygen for 8 hours) (Fig. 3, A and B), indicating that increased CD59 endocytosis is specific to hypoxia/reoxygenation.

### Endothelial deposition of MAC is increased in IH

To investigate whether reduced plasma membrane CD59 observed in OSA has functional consequences, we assessed deposition of MAC on HUVECs in IH. After incubation with 20% normal human serum (serving as a source of complement components), IH increased deposition of MAC on HUVECs by 3.7-fold compared with normoxic condition (Fig. 3C). In parallel with the lack of effect on CD59 endocytosis, continuous hypoxia did not affect deposition of MAC on HUVECs (Fig. 3C), indicating that diminished protection against complement activation is specific to hypoxia/reoxygenation. Amounts of circulating MAC were similar in the serum of OSA patients (*n* = 30) and controls (*n* = 27) [enzyme-linked immunosorbent assay (ELISA); 6.08 ± 6.59 versus 8.09 ± 7.01; two-sided Student's *t* test adjusted for age, gender, and BMI (fig. S2), *P* = NS]. However, MAC deposition was greater in ECs of OSA patients compared with controls (two-sided exact permutation test, *P* = 0.006) (Fig. 3D), suggesting that protection against complement activity is impaired in the endothelium of OSA patients.

### Enhanced endothelial MAC deposition promotes inflammation in IH

**Fig. 4:**
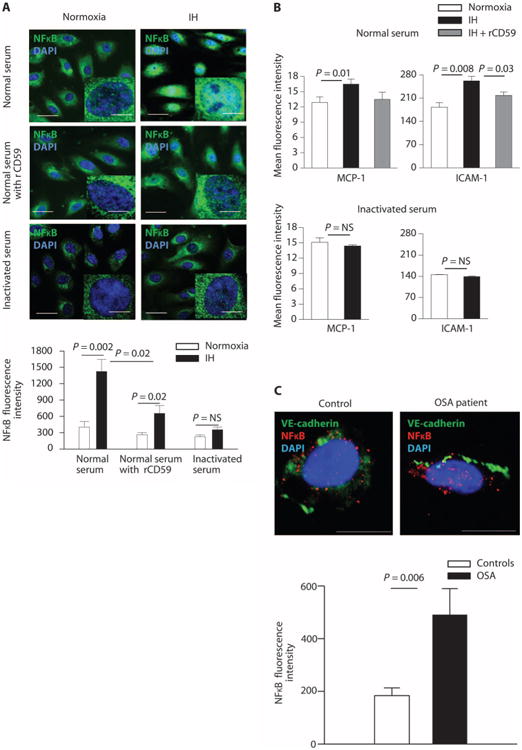
IH promotes endothelial inflammation in a CD59-dependent manner (**A**) Representative confocal images showing nuclear translocation of NFκB in HUVECs exposed to normoxia or IH and incubated with 20% normal serum before and after addition of recombinant CD59 and with heat-inactivated serum. Scale bars, 10 μm; inset, 5 μm. Bar graph quantitates NFκB fluorescence intensity in HUVECs (*n* = 9). (**B**) Quantitation of the mean fluorescence of MCP-1 and ICAM-1 in permeabilized HUVECs incubated with 20% normal serum with or without recombinant CD59, or with heat-inactivated serum in normoxia and IH (flow cytometry; *n* = 5). (**C**) Representative confocal images of NFκB fluorescence intensity in ECs of OSA patients and controls. Scale bars, 10 μm. Bar graph quantitates NFκB fluorescence intensity in ECs from OSA patients (*n* = 9: 55% male; age, 42.4 ± 4.5 years; BMI, 34.5 ± 3.4 kg/m^2^; AHI, 8.2 ± 1.6 events/hour; ODI, 5.4 ± 1.8 events/hour) and controls (*n* = 7:14% male; age, 43.9 ± 5.0 years; BMI, 36.0 ± 4.9 kg/m^2^; AHI, 1.4 ± 0.7 events/hour; ODI, 0.9 ± 0.9 events/hour). Linear regression confirmed that the difference in NFκB fluorescence intensity in ECs among groups is not confounded by age, gender, or BMI (fig. S2). All data throughout the figure are shown as means ± SE (two-sided exact permutation test).

Deposition of MAC on nucleated cells increases nuclear translocation of nuclear factor κB (NFκB) and activation of proinflammatory genes (22, 23). We assessed whether diminished protection against complement activity in IH affects endothelial inflammation. Nuclear translocation of NFκB and expression of proinflammatory cytokines monocyte chemoattractant protein–1 (MCP-1) and intercellular adhesion molecule–1 (ICAM-1) were increased in HUVECs exposed to IH and normal serum compared with normoxia (Fig. 4, A and B), whereas expression of vascular cell adhesion molecule–1 (VCAM-1) was not affected (fluorescence intensity by flow cytometry; mean ± SE, 7.5 ± 0.5 versus 8.0 ± 0.6 for normoxia and IH, respectively; two-sided exact permutation test, *P* = NS). After addition of recombinant CD59, which binds competitively to C8 and C9, thereby reducing MAC formation, nuclear translocation of NFκB was reduced. Expression of MCP-1 and ICAM-1 was similar in normoxia and IH in the presence of recombinant CD59 (Fig. 4, A and B). To investigate whether IH promotes endothelial inflammation through complement activity, we also exposed HUVECs to IH in the presence of serum inactivated by heat, which renders serum free of complement components. Nuclear translocation of NFκB and the expression of MCP-1 and ICAM-1 were similar in normoxia and IH in the absence of complement-containing serum, suggesting that IH promotes endothelial inflammation predominantly via complement (Fig. 4, A and B). Nuclear fluorescence intensity of NFκB was greater in ECs of OSA patients compared with controls (two-sided exact permutation test, *P* = 0.006) (Fig. 4C), thereby confirming that OSA is associated with endothelial inflammation.

### Statins prevent CD59 internalization

**Fig. 5:**
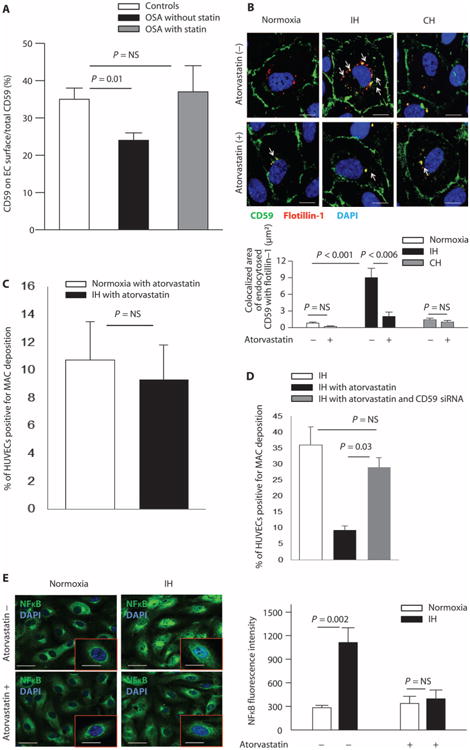
Statins prevent IH-induced internalization of CD59 and MAC deposition on ECs in CD59-dependent manner (**A**) Quantitation of the percentage of total endothelial CD59 located on the plasma membrane in OSA patients taking statins (*n* = 10 controls not taking statins: 60% male; age, 38.4 ± 3.4 years; BMI, 29.4 ± 1.6 kg/m^2^; AHI, 1.9 ± 0.7 events/hour; ODI, 1.1 ± 0.6 events/hour; *n* = 9 OSA patients not taking statins: 33% male; age, 48.7 ± 3.0 years; BMI, 34.9 ± 2.0 kg/m^2^; AHI, 19.4 ± 5.8 events/hour; ODI, 12.3 ± 3.3 events/hour; *n* = 5 OSA patients taking statins: 60% male; age, 56.4 ± 4.6 years; BMI, 32.6 ± 2.2 kg/m^2^; AHI, 16.2 ± 2.3 events/hour; ODI, 8.5 ± 4.3 events/hour). Linear regression confirmed that the difference in plasma membrane expression of CD59 among groups is not confounded by age, gender, or BMI (fig. S2). (**B**) Representative confocal images of CD59 endocytosis in HUVECs in normoxia, IH, and continuous hypoxia without and with atorvastatin (*n* = 4). Arrows indicate endocytosed CD59 with flotillin (colocalized area in μm^2^). Scale bars, 10 μm. Bar graph quantitates colocalized area of endocytosed CD59 with flotillin-1. (**C**) Quantitation of the percentage of HUVECs positive for MAC deposition in normoxia and IH after incubation with 20% normal serum and atorvastatin (*n* = 4; flow cytometry). (**D**) Quantitation of the percentage of HUVECs positive for MAC deposition in IH after incubation with 20% normal serum with and without atorvastatin, and with or without transfection with CD59 siRNA to achieve CD59 knockdown (*n* = 4; flow cytometry). (**E**) Representative confocal images of nuclear translocation of NFκB in HUVECs exposed to normoxia or IH and incubated with 20% normal serum with or without atorvastatin. Bar graph quantitates NFκB fluorescence intensity in HUVECs in normoxia and IH with and without atorvastatin (*n* = 4). Scale bars, 20 μm; inset, 10 μm. All data throughout the figure are shown as means ± SE (two-sided exact permutation test).

In the subset of OSA patients who were receiving statin therapy, the percentage of total CD59 located on the plasma membrane was similar to controls and significantly greater than in their OSA counterparts who were not taking statins (*P* = 0.05), suggesting that statins preserve CD59 on the EC plasma membrane in OSA (Fig. 5A). This unexpected observation led to an investigation of statin effects on CD59 localization and function. HUVECs were incubated with atorvastatin during normoxia or IH. Colocalization of internalized CD59 and flotillin-1 was minimal in IH after atorvastatin treatment and similar to that observed with normoxia and continuous hypoxia, suggesting that atorvastatin abolishes enhanced CD59 endocytosis caused by IH (Fig. 5B). As expected, CD59 did not colocalize with endocytosed transferrin in IH or continuous hypoxia with or without atorvastatin treatment (fig. S3). Deposition of MAC was similar in normoxia and IH in HUVECs incubated with atorvastatin, confirming that statins preserve plasma membrane CD59 in IH (Fig. 5C). After we knocked down *CD59* with small interfering RNA (siRNA) (fig. S4) in HUVECs incubated with atorvastatin in IH, deposition of MAC was similar to IH without statins, indicating a loss of protective effects of atorvastatin on MAC deposition in IH (Fig. 5D). Therefore, the protective effect of atorvastatin against endothelial deposition of MAC in IH is CD59-dependent.

To investigate whether statins affect proinflammatory effects of MAC deposition in IH, HUVECs were incubated with atorvastatin and normal serum. Atorvastatin reduced nuclear translocation of NFκB in IH (Fig. 5E). Thus, statins stabilize plasma membrane localization of endothelial CD59 and reduce IH-induced endothelial MAC deposition and consequent inflammation.

### IH reduces expression of cholesterol efflux–promoting genes

**Fig. 6:**
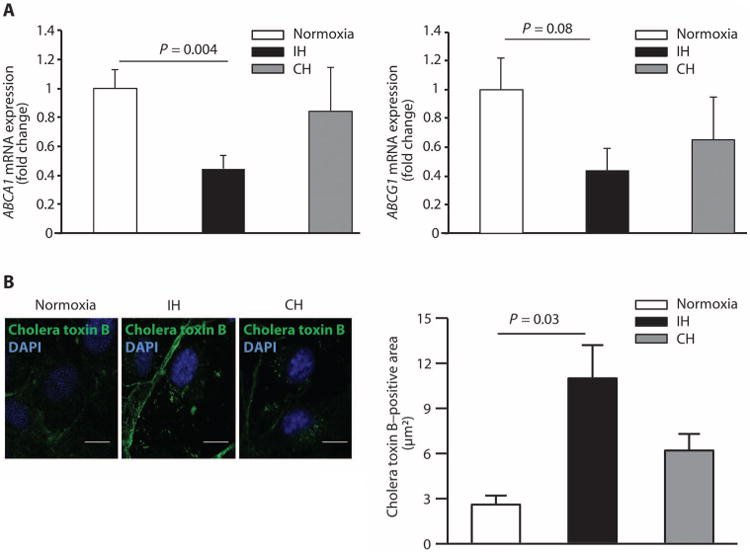
IH increases cellular cholesterol by decreasing gene expression of cholesterol efflux mediators (**A**) Quantitation of the *ABCA1* and *ABCG1* mRNA expression in HUVECs in normoxia, IH, and continuous hypoxia expressed as a fold change over normoxia (*n* = 4). (**B**) Representative confocal images of cholera toxin B staining of the plasma membrane (depicting lipid rafts containing free cholesterol) in HUVECs in normoxia, IH, and continuous hypoxia. Scale bars, 10 μm. Bar graph quantitates cholera toxin B–positive area (μm^2^) (*n* = 4). All data throughout the figure are shown as means ± SE (two-sided exact permutation test).

To investigate the mechanisms that mediate enhanced endocytosis of CD59 in IH and the protective effects of statins, we quantified the expression of genes that regulate endothelial cholesterol efflux and synthesis. Increased cellular cholesterol results in more abundant lipid rafts and enhanced endocytosis of GPI-anchored proteins, which reside in lipid rafts (24). Prolonged continuous hypoxia (48 hours) promotes cellular accumulation of cholesterol by reducing its efflux via adenosine triphosphate–binding cassette subfamily A member 1 (ABCA1) and ABCG1 cassette transporters in macrophages (25). Expression of *ABCA1* and *ABCG1* mRNA in HUVECs was reduced by 60%, and cholera toxin B staining of the plasma membrane lipid rafts was more prominent in IH compared with normoxia, whereas 8 hours of continuous hypoxia had no significant effect (Fig. 6, A and B), suggesting that IH increases cholesterol-enriched membrane domain formation in ECs. Although the mRNA expression of *3-hydroxy-3-methylglutaryl-coenzyme A (HMG-CoA) reductase and synthase*, key enzymes in cholesterol biosynthesis, was not altered by IH (fig. S5), statins may reduce cholesterol biosynthesis and thus reverse increased cholesterol-enriched plasma membrane domain formation and endocytosis of endothelial CD59, thereby preserving protection against complement activity in OSA.

## Discussion

Using ECs harvested from OSA patients and a phage display library, we have identified a role for complement regulator CD59 in OSA. Although we discovered that the overall amount of CD59 is increased in ECs from OSA patients, its cellular distribution is also markedly altered, with a relative decrease of cell surface CD59 and an increased intracellular abundance. We used HUVECs to confirm that IH promotes endocytosis of CD59, decreasing protection against complement activity. Consequently, deposition of MAC on ECs is increased in IH, resulting in endothelial inflammation. Because the expression of CD59 is up-regulated by NFκB activation (26–28), our results suggest that IH promotes internalization of CD59 in ECs of OSA patients, causing increased MAC deposition and NFκB activation and eventually a compensatory increase in CD59 expression in an attempt to restore protection against complement activity. However, such restoration of homeostasis appears to be achieved at the expense of increased NFκB activation with its attendant adverse effects on EC function (10). Our data suggest that the abnormal cycle of increased internalization of CD59 and inflammation in IH can be disrupted by statin therapy, potentially uncovering a protective effect of statins on vascular function in OSA patients.

The complement pathway has not been previously linked to increased cardiovascular risk in OSA; however, CD59 deficiency promotes MAC deposition and accelerates atherosclerosis in *ApoE*^−/−^ and *Ldlr*^−/−^ mice (29, 30), whereas selective overexpression of human *CD59* in the endothelium and hematopoietic cells renders *ApoE*^−/−^ mice resistant to atherogenesis (31). Deposition of MAC stimulates an inflammatory response, including increased nuclear translocation of NFκB and expression of proinflammatory genes, which promote atherosclerosis (22, 23, 26). Increased NFκB activity and expression of proinflammatory cytokines have been noted in OSA without a clear understanding of the underlying mechanisms (10, 32). Our finding that IH-induced endocytosis of CD59 causes MAC deposition on ECs and inflammation links complement activation to vascular inflammation in OSA. MAC-induced nuclear translocation of NFκB is the main regulator of inducible expression of CD59, which likely underlies the observed compensatory trend toward up-regulation of *CD59* gene expression to protect cells from complement-mediated injury and greater intracellular abundance of CD59 protein in OSA patients (26–28). However, this new “steady state” is accompanied by increased NFκB-mediated inflammation, which may be detrimental to endothelial health in OSA. IH did not substantially affect NFκB translocation or proinflammatory cytokine expression in ECs in the absence of complement, suggesting that complement activation and decreased complement inhibition are predominant contributors to vascular inflammation in conditions associated with IH, such as OSA.

Whereas children with a rare homozygous loss of function of *CD59* in hematopoietic cells suffer from thromboembolic events, hemolysis, and peripheral neurologic disease before the age of 5 years, their heterozygous parents are asymptomatic. The heterozygous parents were not followed longitudinally, thereby precluding definitive conclusions regarding the effects of heterozygous loss of function of *CD59* on cardiovascular risk. Chronically increased internalization of endothelial CD59, which appears to occur in untreated OSA before the onset of clinically evident cardiovascular disease, may underlie chronic endothelial inflammation that likely contributes to the well-documented increased cardiovascular risk in OSA (33–37).

GPI-anchored proteins, including CD59, are internalized by a clathrin-independent pathway that is cholesterol-dependent (38). Filipin, which traps cholesterol, selectively inhibits endocytosis of GPI-anchored proteins but not clathrin cargo, indicating that free cholesterol in the membrane is required for GPI-anchored protein endocytosis (24). IH increased free cholesterol content in EC plasma membrane, likely by reducing gene expression of cholesterol efflux mediators ABCA1 and ABCG1, which may underlie the increased endocytosis of CD59. Mice deficient in the p50 subunit of NFκB on a high-cholesterol diet are protected from IH-induced ABCA1 inhibition and accelerated atherosclerosis, suggesting that ABCA1 and ABCG1 inhibition in IH may be mediated by NFκB activation (39).

Currently, no targeted therapy is available for vascular manifestations of OSA. Our unexpected finding that statins reduce complement-related vascular inflammation in OSA suggests a therapeutic strategy for OSA-related vascular risk that may be complementary to CPAP. Statins also have antioxidant effects, which may be particularly beneficial in conditions associated with oxidative stress such as OSA (40). Statin effects on CD59 in IH appear to be cholesterol-dependent. Decreased cholesterol influences plasma membrane sorting of GPI-anchored proteins and raft-mediated endocytosis (41). Our finding that atorvastatin preserves cell surface CD59 corroborates previous reports that atorvastatin increases endothelial expression of CD59 (by inhibiting geranylgeranylation) and another GPI-anchored protein, ecto-5′-nucleotidase (by inhibiting isoprenylation of Rho–guanosine triphosphatases), in the setting of prolonged continuous hypoxia (18 to 72 hours) (42, 43). Statin-induced reduction of cholesterol results in decreased endocytosis of CD59 and protects ECs from MAC deposition in IH and OSA. Thus, in addition to lowering LDL cholesterol and acting as antioxidants, statins may exert endothelial protection in OSA by inhibiting complement activation.

The main limitation of this study is that we searched for possible pathways that activate the endothelium in OSA by profiling venous ECs harvested from OSA patients. The precise mechanisms underlying cardiovascular risk in OSA cannot be ascertained from the study of the venous endothelium. Local biomechanical forces that affect arterial ECs at specific sites play an essential role in determining regional susceptibility to atherosclerosis, and endothelial biopsy at specific sites of the arterial vasculature will likely be required to determine the precise mechanisms underlying atherosclerosis in OSA. However, venous and arterial ECs are exposed to the same circulating proinflammatory factors and hypoxia/reoxygenation in OSA. Inflammatory and oxidative pathways are activated similarly in venous and arterial ECs in healthy subjects and patients with atherosclerosis (44, 45). Furthermore, the culturing process rapidly erases arteriovenous gene expression differences that are present in freshly isolated ECs (46). Thus, direct investigation of easily accessible freshly isolated human venous ECs without artifact of culture condition may be particularly useful in conditions associated with systemic endothelial activation, such as OSA (2, 5, 6). Another limitation is the duration of hypoxic episodes. For in vitro experiments, we used repetitive 30-min episodes of hypoxia, which differ from the duration of the hypoxic episodes in OSA patients that typically last 20 to 40 s. This was due to the time required to achieve the set level of hypoxia in the hypoxic chamber, which is slightly longer than the typical hypoxic episode in OSA, and to ensure sufficient exposure to IH during in vitro experiments. An additional limitation of our study is that some OSA patients had undergone a split-night polysomnography study, which includes CPAP titration; however, all patients had at least 2 hours of baseline polysomnographic recording, and only those with at least 30 obstructive events during the baseline recording underwent split-night CPAP titration, thereby ensuring that OSA was well documented.

In conclusion, repetitive IH, a hallmark of OSA, promotes internalization of complement inhibitor CD59 and consequent MAC deposition on ECs, increasing nuclear translocation of NFκB. The resulting chronic endothelial inflammation may contribute to increased cardiovascular risk in OSA. Internalization of CD59 appears to be cholesterol-dependent, and MAC deposition on ECs in IH is reversed by statins in a CD59-dependent manner, suggesting a possible therapeutic strategy to reduce vascular risk in OSA.

## Materials and Methods

### Study design

We prospectively recruited participants from the Sleep Disorders Center at Columbia University. All study participants underwent nocturnal polysomnography. An AHI ≥5 obstructive events/hour of sleep established the diagnosis of OSA. Study participants with AHI <5 events/hour were considered OSA-free. All patients with suspected sleep-disordered breathing who were referred for nocturnal polysomnography were screened. Patients with coronary artery disease, heart failure, a history of stroke, diabetes mellitus, chronic obstructive or restrictive pulmonary disease, chronic kidney disease, or tobacco use within the past 10 years were ineligible for the study. The Columbia University Committee on Human Research approved the study. All study participants gave written informed consent. There was no prespecified effect size in this prospective study. Post hoc power analysis showed that when statistical significance was reported for human samples, the probability of rejecting the null hypothesis was >90%. No samples were excluded from the study. Experiment endpoints were defined prospectively. The number of replicates in each experiment is stated individually for each experiment. The prespecified hypothesis defining the research objectives was that repetitive hypoxia/reoxygenation that characterizes OSA activates the endothelium in a distinctive manner. Nocturnal polysomnography was performed with an Embla signal recording system and Somnologica Window NT Software (Flaga Group hf. Medical Devices) as previously described (14). Briefly, sleep stages were scored in 30-s epochs according to standard criteria. An obstructive apnea was defined as a cessation of upper airway flow in association with continued respiratory effort of at least 10 s. An obstructive hypopnea was defined as a discrete reduction in airflow associated with a decrease in oxygen saturation of 2% or more for at least 10 s in the presence of thoracoabdominal ventilatory efforts. AHI was defined as the number of obstructive apnea plus hypopnea episodes per hour of sleep. An ODI was defined as the number of ≥3% arterial oxygen desaturations/hour of sleep. Daytime sleepiness was assessed by Epworth sleepiness scale (0 to 9, normal sleepiness; 10 to 24, increased sleepiness) (47). All study participants had at least 2 hours of a baseline polysomnographic recording. EC harvesting was performed between 9:00 and 11:00 a.m. after polysomnography while study participants were in a fasting state as described previously (14). In OSA patients who underwent split-night polysomnography with CPAP titration, harvesting was performed 48 hours later to avoid any CPAP interference.

### EC harvesting and isolation

A 20-gauge angiocatheter was inserted into a superficial forearm vein. Under sterile conditions, three J-shaped vascular guide wires (Arrow) were sequentially advanced into the vein up to 10 cm. ECs were retrieved from wire tips by washing with EC dissociation buffer. Harvesting yielded ∼2000 to 5000 ECs. For immunofluorescence, ECs were recovered by centrifugation at 4°C, 150*g* for 6 min, and then the cell pellet was resuspended in red blood cell (RBC) lysis buffer (eBioscience), incubated at 4°C for 5 min, centrifuged at 150*g* for 6 min, fixed with 4% paraformaldehyde (Santa Cruz Biotechnology) in phosphate-buffered saline (PBS) for 10 min, washed twice with PBS, transferred to poly-l-lysine–coated slides (Sigma), and then air-dried at 37°C. The slides were stored at −80°C until analyzed. For phage display and mRNA extraction, the cell pellet was resuspended in isolation buffer, incubated with biotinylated mouse anti-human monoclonal antibody directed against CD146 (1:200; catalog no. MAB16985B, Millipore) at 4°C for 15 min, incubated at 4°C with Streptavidin FlowComp Dynabeads (1:100; Invitrogen) for 45 min, and then subjected to EC isolation by magnet. For flow cytometry, the cell pellet was resuspended in RBC lysis buffer, incubated at 4°C for 5 min, and then centrifuged at 150*g* for 6 min. The isolated cell pellet was resuspended in PBS.

### Phage display library

Panning, or in vitro selection binding, was performed by incubating a library of 2 × 10^11^ M13 phage–displayed seven–amino acid peptides (New England Biolabs) with purified harvested ECs on 96-well polystyrene plates according to the manufacturer's protocol. After 2 hours of incubation at room temperature, unbound phage was washed away and EC-bound phage was eluted by lowering the pH. Eluted phage was then amplified by *Escherichia coli* [mid-log phase *E. coli* ER2738; OD_600_ (optical density at 600 nm) ≈ 0.5]. Aliquots of the bacterial culture and phage mixture were plated onto LB agar plates containing 5-bromo-4-chloro-3-indolyl-β-**D**-galactoside (X-Gal) and isopropyl-β-d-thiogalactopyranoside (IPTG) and incubated overnight at 37°C. Two additional amplification cycles were performed to enrich the pool of selected phages. After three amplification cycles, 24 random plaques were transferred to diluted *E. coli* culture for additional amplification. Only plaques from plates containing <100 plaques were used to ensure that each plaque contains a single phage–derived DNA insert. Samples were purified and sequenced at the Columbia University Proteomics Core Facility. For positive control, panning was performed using streptavidin as the target. Bound phage was eluted with 0.1 mM biotin in tris-buffered saline for 30 min. After three amplification cycles, the consensus sequence for streptavidin-binding peptides was S/T/N L L/I/V A/N HPQ, with the HPQ motif being the most prominent (18). For negative control, an identical panning protocol was performed without target cells in 20 μl of NaHCO_3_ and bovine serum albumin on 96-well polystyrene plates. Isolated phages from the negative control experiment are shown in table S2.

### Cell culture

HUVECs (BD Biosciences) were cultured in EC medium with 2% endotoxin-free heat-inactivated fetal bovine serum (FBS; Life Technologies) until they reached the required confluence for each experiment. During 8 hours of normoxia, IH, or continuous hypoxia, HUVECs were incubated with either 20% normal human serum or heat-inactivated serum (Sigma-Aldrich) as detailed for each experiment. For atorvastatin experiments, HUVECs were incubated with 20% normal serum with or without addition of 1 μM atorvastatin (Sigma-Aldrich) for 8 hours in normoxia, IH, or continuous hypoxia. Passages 3 to 5 were used for all experiments. HEK293 [American Type Culture Collection (ATCC)] were cultured in Eagle's minimum essential medium with 10% FBS (ATCC).

### Immunofluorescence

For confirmation of the binding of peptide F to harvested ECs, unpermeabilized ECs were incubated with 2 μg of peptide F (Proteintech Group) for 2 hours at 4°C. After washing three times with PBS and blocking with PBS containing 5% donkey serum (Sigma-Aldrich), ECs were incubated with rabbit polyclonal antibodies directed against peptide F (1:500; Proteintech), followed by Texas Red–conjugated donkey anti-rabbit secondary antibodies (1:50; Jackson ImmunoResearch Laboratories). Appropriate negative control slides were generated without either peptide F or anti–peptide F antibody. Nuclei were stained with 4′6-diamidino-2-phenylindole (DAPI; Molecular Probes by Life Technologies). ECs were identified by positive staining with mouse anti-human antibodies directed against von Willebrand factor (1:50; DAKO), followed by FITC-conjugated donkey anti-mouse secondary antibodies (1:50; Jackson ImmunoResearch). For assessment of cellular distribution of CD59, harvested ECs were identified by positive staining with goat anti-human polyclonal antibodies directed against CD144 (VE-cadherin) (1:50; R&D Systems) followed by FITC-conjugated donkey anti-goat secondary antibodies (1:50; Jackson ImmunoResearch). CD59 expression was assessed with rabbit anti-human antibodies directed against CD59 (1:50; Abcam) followed by Texas Red–conjugated donkey anti-rabbit secondary antibodies (1:50; Jackson ImmunoResearch). ECs were analyzed with a confocal microscope (Nikon A1, Nikon Instruments Inc.). The threshold for positive staining was set at 40% of maximum fluorescence intensity. We used ImageJ to draw two lines along the EC plasma membrane, which was defined as the area with positive staining for VE-cadherin. The inner line was drawn along the interior border of VE-cadherin–positive area, and the outer line was drawn along the exterior border of VE-cadherin–positive area. The fluorescence intensity of the CD59-positive area between two lines was quantified (μm^2^ ) and represented EC surface CD59 expression. The fluorescence intensity of the CD59-positive area in each EC located within an outer line represented total EC CD59 expression (μm^2^). The ratio of the CD59-positive area between two lines divided by the CD59-positive area located within an outer line (total EC CD59 expression) represented the proportion of CD59 located on the EC surface. At least 25 consecutive ECs were analyzed for each slide. Investigators who analyzed the slides were blinded to the OSA status of the study participant. To assess endocytosis of CD59, HUVECs that were grown to 70 to 80% confluence were exposed to normoxia, IH, or continuous hypoxia for 8 hours with or without 1 μM atorvastatin. During the last 5 min, cells were incubated with primary antibodies directed against CD59 (mouse anti-human,1:50; BD Biosciences) and flotillin-1 (rabbit anti-human, 1:100; Thermo Scientific) or transferrin (rabbit anti-human, 1:100; Thermo Scientific) at 37°C for 5 min, washed, and then fixed with 4% paraformaldehyde at room temperature for 10 min. Cells were then permeabilized with 0.1% Triton X-100 (Acros Organics), washed, and incubated with FITC-conjugated donkey anti-mouse (1:50; Jackson ImmunoResearch) and Texas Red–conjugated donkey anti-rabbit (1:50; Jackson ImmunoResearch) secondary antibodies at room temperature for 30 min, washed with PBS, and stained with DAPI. We used ImageJ software for colocalization analysis. Colocalization was expressed in μm^2^. For assessment of MAC deposition on harvested ECs, unpermeabilized harvested ECs were identified by positive staining with goat anti-human polyclonal antibodies directed against CD144 (VE-cadherin) (1:50; R&D Systems), followed by FITC-conjugated donkey anti-goat secondary antibodies (1:50; Jackson ImmunoResearch). MAC deposition was assessed with mouse anti-human monoclonal antibodies directed against C5b-9 (MAC) (clone aE11, 1:10; DAKO), followed by Texas Red–conjugated donkey anti-mouse secondary antibodies (Jackson ImmunoResearch; 1:50), and analyzed with a fluorescence microscope (Nikon Eclipse E600). For assessment of NFκB activation in vitro, HUVECs were permeabilized with 0.1% Triton X-100 and incubated with rabbit anti-human antibodies directed against the p65 subunit of NFκB (1:50; Novus), followed by FITC-conjugated donkey anti-rabbit secondary antibodies (1:50; Jackson ImmunoResearch), and stained with DAPI. For assessment of NFκB activation in harvested ECs, permeabilized harvested ECs were identified by positive staining with goat anti-human polyclonal antibodies directed against CD144 (VE-cadherin) (1:50; R&D Systems), followed by FITC-conjugated donkey anti-goat secondary antibodies (1:50; Jackson ImmunoResearch). Nuclear fluorescence of NFκB was assessed by confocal microscopy after staining with rabbit anti-human antibodies directed against the p65 subunit of NFκB (1:50; Novus), followed by Texas Red–conjugated donkey anti-rabbit secondary antibodies (1:50; Jackson ImmunoResearch), and stained with DAPI. For assessment of cholesterol-enriched membrane domains, HUVECs were permeabilized with 0.1% Triton X-100 and incubated with cholera toxin subunit B conjugated with Alexa Fluor 488 (1:100; Molecular Probes by Life Technologies) and stained with DAPI. All HUVECs prepared for immunofluorescence were analyzed by confocal microscopy (Nikon A1).

### Native gel and mass spectrometry

HUVEC whole-cell lysate (100 μg was incubated with 30 μg of peptide F (CFHENWPS, Proteintech) at 4°C for 2 hours. The mixture was diluted with 2× native sample buffer (Bio-Rad) and loaded on a 10% native gel in running buffer without SDS [NativePAGE Running Buffer (20×), Thermo Scientific]. The gel was transferred to a nitrocellulose membrane and probed with antibodies directed against peptide F (rabbit polyclonal antibody, 1:500; Proteintech). A duplicate gel was stained with GelCode Blue Stain Reagent (Thermo Scientific). The corresponding band of interest was cut and sent to Columbia University Protein Chemistry Core Facility for mass spectrometry.

### In vitro interaction of peptide F with recombinant CD59

Peptide F (2 μg) (CFHENWPS, Proteintech) and control peptide (2 μg) (CPLAYGTW, Proteintech) were incubated separately with 1 μg of recombinant CD59 (R&D Systems) in 21 μl of PBS on a slow shaker overnight at 4°C. The cross-linking reagent 3,3′-dithiobis[sufosuccinimidylpropionate] (DTSSP) (Thermo Scientific) was added to a final concentration of 2 mM. After rotation at room temperature for 1 hour, the samples were diluted in 2× sample buffer (Bio-Rad) with β-mercapto-ethanol and loaded on 15% SDS-PAGE.

### Transfection of HEK293 cells with Myc-DDK–tagged human CD59 plasmid

HEK293 cells were transfected with 5 μg of (Myc-DDK-tagged)-Human CD59 plasmid (OriGene) and 2.5 μl of EndoFectin Plus transfection reagent (GeneCopoeia) in a confluent 35-mm plate according to the manufacturer's protocol. After transfection for 72 hours, the HEK293 cells were lysed with immunoprecipitation (IP) lysis buffer (Thermo Scientific). The expression of Myc-DDK–tagged human CD59 was confirmed by detecting DDK with anti-DDK monoclonal antibody (OriGene) by Western blotting.

### Immunoprecipitation

Transfected HEK293 cell lysate was incubated with 500 μg of peptide F (Proteintech) or control peptide (Proteintech) overnight at 4°C. The sample was cross-linked with 2 mM DTSSP (Thermo Scientific) for 1 hour at room temperature. The sample was precleared by adding 10 μg of mouse immunoglobulin G and 20 μl of protein A/G agarose and incubated at 4°C for 2 hours. After centrifugation at 1000*g* for 5 min, the supernatant was incubated with 5 μg of anti-DDK antibodies (OriGene) overnight at 4°C. Protein A/G PLUS-Agarose beads (40 μl) (Santa Cruz Biotechnology) were added and incubated for 2 hours at 4°C, then centrifuged for 5 min at 1000*g* at 4°C. The pellet was washed three times with IP lysis buffer (Thermo Scientific), followed by addition of 80 μl of 2× sample buffer (Santa Cruz Biotechnology). The sample was boiled for 5 min and then centrifuged for 5 min at 1000*g* at 4°C. The supernatant was loaded onto a 15% SDS-PAGE gel and probed with antibodies to peptide F (nonreducing condition), DDK, and CD59 (both in reducing condition with 5% β-mercaptoethanol).

### Immunoblotting

HUVECs were lysed on ice for 5 min with lysis buffer (Thermo Scientific) containing 25 mM tris (pH 7.4), 150 mM NaCl, 1% NP-40, 1 mM EDTA, 5% glycerol, and Halt Protease Inhibitor Cocktail (Thermo Scientific). Total amount of protein in the lysate was quantified by protein assay (Bio-Rad) to ensure equal protein loading on gels. Protein samples were separated on 15% SDS-PAGE, followed by immunoblotting with appropriate antibodies.

### Flow cytometry

Harvested EC samples were incubated with RBC lysis buffer at 4°C for 5 min and then centrifuged at 150*g* for 5 min. The pellets were resuspended in 100 μl of PBS. To evaluate CD59 expression, samples were incubated with mouse anti-human antibodies directed against CD59 that were preconjugated with FITC (1:20; eBioscience), mouse anti-human antibodies directed against CD144 (VE-cadherin, 1:20; eBioscience) that were preconjugated with allophycocyanin (APC), and mouse anti-human antibodies directed against CD45 that were preconjugated with phycoerythrin (PE) (1:20; eBioscience) at 4°C for 1 hour. Samples were washed with cold PBS three times, then resuspended with 500 μl of PBS, and analyzed by flow cytometry (BD FACSCalibur). ECs were identified as intact cells positive for CD144/CD59 and negative for CD45. To assess MAC (C5b-9) deposition on HUVECs after 8 hours of exposure to normoxia, IH, or continuous hypoxia, cells were incubated with mouse anti-human antibodies directed against C5b-9 (1:10; DAKO) at 4°C for 1 hour, washed with cold PBS, resuspended with 500 μl of PBS, and analyzed by flow cytometry. Dead cells were identified as positive for propidium iodide staining (Thermo Scientific). For assessment of inflammation, HUVECs collected after trypsinization were incubated with mouse anti-human antibodies directed against MCP-1 (1:20; pre-conjugated with PE), ICAM-1 (1:20; preconjugated with PE), and VCAM-1 (1:20; preconjugated with PE) (all from Abcam) at 4°C for 1 hour, washed with cold PBS, resuspended with 500 μl of PBS, and analyzed by flow cytometry.

### Enzyme-linked immunosorbent assay

Serum was collected from all study participants at the time of EC harvesting, centrifuged at 2000 RCF (relative centrifugal force) for 10 min at 4°C, aliquoted, and stored at −80°C until analyzed. Human terminal complement complex C5b-9 ELISA kit (Novatein Biosciences) was used to quantify the amount of human terminal complement complex C5b-9 (MAC) in the serum according to the manufacturer's protocol. Analysis was performed with a Multiskan EX reader (Thermo Scientific).

### Quantitative real-time PCR

We used TaqMan (Life Technologies) method on Applied Biosystems 7500 Fast Real-Time PCR System. mRNA was extracted using TRIzol (Life Technologies) according to the manufacturer's protocol. mRNA (1 μg) was used for first-strand complementary DNA (cDNA) synthesis with SuperScript III Kit (Life Technologies) according to the manufacturer's protocol. The first strand cDNA was further diluted 10 times with ribonuclease-free water and stored at −80°C. For 20 μl of realtime PCRs, the following reagents were added: 10 μl of TaqMan Gene Expression Master Mix (2×) (Life Technologies), 1 μl of TaqMan Gene Expression Assay (20×), 5 μl of nuclease-fee water, and 4 μl of cDNA. This reaction mix was added to the wells as duplicates for each sample. Real-time PCR products were run on a 1% agarose gel to confirm the size of the products. All the real-time PCR primers and probes were designed to span an exon junction to eliminate the contamination of genomic DNA. TaqMan primers and probes were used for *CD59*, β-*actin*, *HMG-CoA synthase and reductase*, *ABCG1*, and *ABCA1*. Results were expressed as *C*_t_ values normalized to the housekeeping gene β-*actin*.

### Intermittent and continuous hypoxia exposure

We used a hypoxia chamber (BioSpherix) housed inside an incubator kept at 37°C for all hypoxia experiments. HUVECs subjected to IH were exposed to 21% O_2_ with 5% CO_2_ for normoxia and 2% O_2_ with 5% CO_2_ for hypoxia for alternating 30-min cycles for a total exposure time of 8 hours. For continuous hypoxia, HUVECs were exposed to 2% O_2_ with 5% CO_2_ for 8 hours.

### Transfection and siRNA CD59 treatment of HUVECs

siRNA treatment was carried out with Lipofectamine RNAiMax (Life Technologies) for 48 hours according to the manufacturer's protocol using 50 nM oligonucleotide. The specific oligonucleotides were directed at human *CD59* (AGCCGUCAAUUGUUCAUCUtt) (siRNA ID s2696, s2698, or combination of s2696 and s2698; Life Technologies). Two scramble siRNAs were used as a negative control, and siRNA against glyceraldehyde-3-phosphate dehydrogenase (GAPDH) (Life Technologies) was used as a positive control. Successful transfection was confirmed by >90% reduction in CD59 protein expression in three independent experiments using Western blotting. Because the expression of CD59 was almost completely knocked down by either siRNA (s2696 or s2698), s2698 was arbitrarily selected for subsequent experiments.

### Statistical analysis

Results are presented as means ± SE or percentage (%), as appropriate. The baseline characteristics of OSA patients and control subjects are expressed as the group-specific summary statistics, including age, gender, BMI, arterial oxyhemoglobin saturation (SaO_2_) nadir, AHI, ODI, time spent below SaO_2_ of 90% during sleep (*t* <90%), Epworth sleepiness scale, systolic and diastolic blood pressure, and physician-confirmed diagnosis of hypertension and dyslipidemia. Whether baseline characteristics significantly differ between the two groups was formally tested by two-sample *t* test (for continuous variables) or χ^2^ test (for binary variables). Two-sample *t* test was used for comparisons of mean values of peptide F frequency and the expression of mRNA and total and plasma membrane CD59 protein between OSA patients and controls (48). For the expression of plasma membrane CD59 protein in OSA patients receiving statin therapy, OSA patients who were or were not receiving statin therapy were compared with controls using two-sample *t* test. For sensitivity analysis, these comparisons were repeated using Wilcoxon rank tests and exact permutation tests (48, 49). A linear model for each outcome was constructed, with OSA status as the primary predictor and age, BMI, and gender as covariates. We used linear models to evaluate whether these covariates are associated with the outcome and whether the observed differences between OSA patients and controls are confounded by age, BMI, and/or gender. *P* values of <0.05 indicate statistical significance. No statistical method was used to predetermine sample size. Because of the small sample size in experiments using cultured cells, two-sided exact permutation test was used for all comparisons (49).

## Supplementary Material

supplement www.sciencetranslationalmedicine.org/cgi/content/full/8/320/320ra1/DC1
Fig. S1. Expression of CD59 in leukocytes and platelets.
Fig. S2. Linear regression analysis.
Fig. S3. Endocytosis of CD59 with transferrin.
Fig. S4. Confirmation of HUVEC transfection with CD59 siRNA.
Fig. S5. Cholesterol biosynthesis in IH.
Table S1. Baseline characteristics of patients with OSA and control subjects.
Table S2. Phages isolated after panning without target cells and with bovine serum albumin in polystyrene wells.
