# Supplementary material for: Increased internalization of complement inhibitor CD59 may contribute to endothelial inflammation in obstructive sleep apnea
Source: Sci Transl Med. Author manuscript; Available in PMC 2017 Jun 27. (PMC5485919; doi:10.1126/scitranslmed.aad0634)
Supplement: supplement — www.sciencetranslationalmedicine.org/cgi/content/full/8/320/320ra1/DC1 Fig. S1. Expression of CD59 in leukocytes and platelets. Fig. S2. Linear regression analysis. Fig. S3. Endocytosis of CD59 with transferrin. Fig. S4. Confirmation of HUVEC transfection with CD59 siRNA. Fig. S5. Cholesterol biosynthesis in IH. Table S1. Baseline characteristics of patients with OSA and control subjects. Table S2. Phages isolated after panning without target cells and with bovine serum albumin in polystyrene wells. [file NIHMS867684-supplement-supplement.pdf]

Science Translational Medicine AAAS

www.sciencetranslationalmedicine.org/cgi/content/full/8/320/320ra1/DC1

Supplementary Materials for

Increased internalization of complement inhibitor CD59 may contribute to endothelial inflammation in obstructive sleep apnea

Memet Emin, Gang Wang, Francesco Castagna, Josanna Rodriguez-Lopez, Romina Wahab, Jing Wang, Tessa Adams, Ying Wei, Sanja Jelic*

*Corresponding author. E-mail: sj366@cumc.columbia.edu

Published 6 January 2016, *Sci. Transl. Med.* **8**, 320ra1 (2016)
DOI: 10.1126/scitranslmed.aad0634

The PDF file includes:

Fig. S1. Expression of CD59 in leukocytes and platelets.
Fig. S2. Linear regression analysis.
Fig. S3. Endocytosis of CD59 with transferrin.
Fig. S4. Confirmation of HUVEC transfection with CD59 siRNA.
Fig. S5. Cholesterol biosynthesis in IH.
Table S1. Baseline characteristics of patients with OSA and control subjects.
Table S2. Phages isolated after panning without target cells and with bovine serum albumin in polystyrene wells.

Supplementary Materials

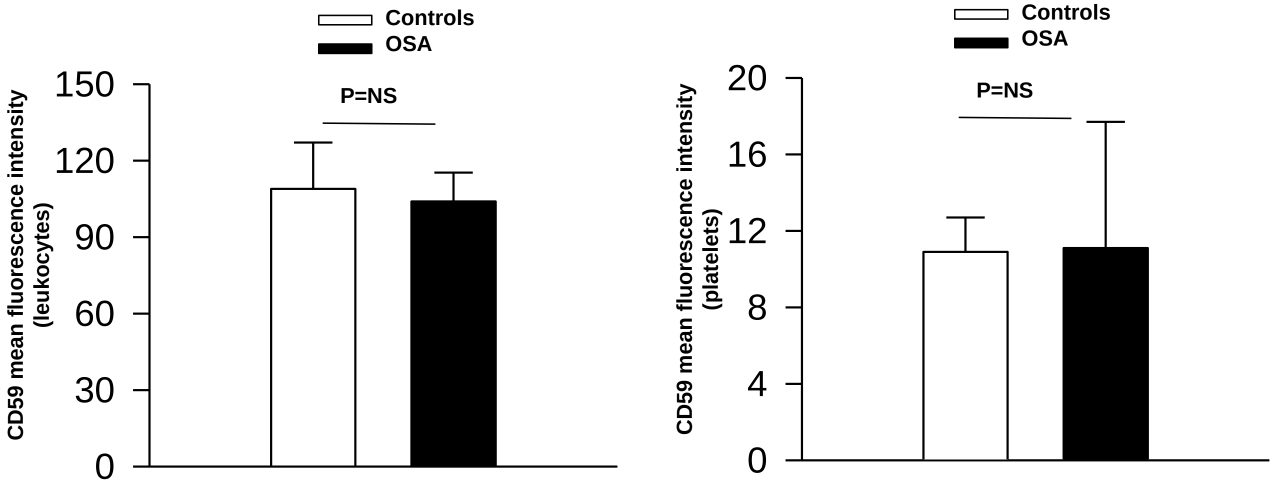

Fig. S1. Expression of CD59 in leukocytes and platelets. Bar graphs quantitate the expression of CD59 in unpermeabilized leukocytes and platelets of OSA patients (n=7) and controls (n=7) (flow cytometry, mean ± SE, 2-sided Exact permutation test). NS=not significant.

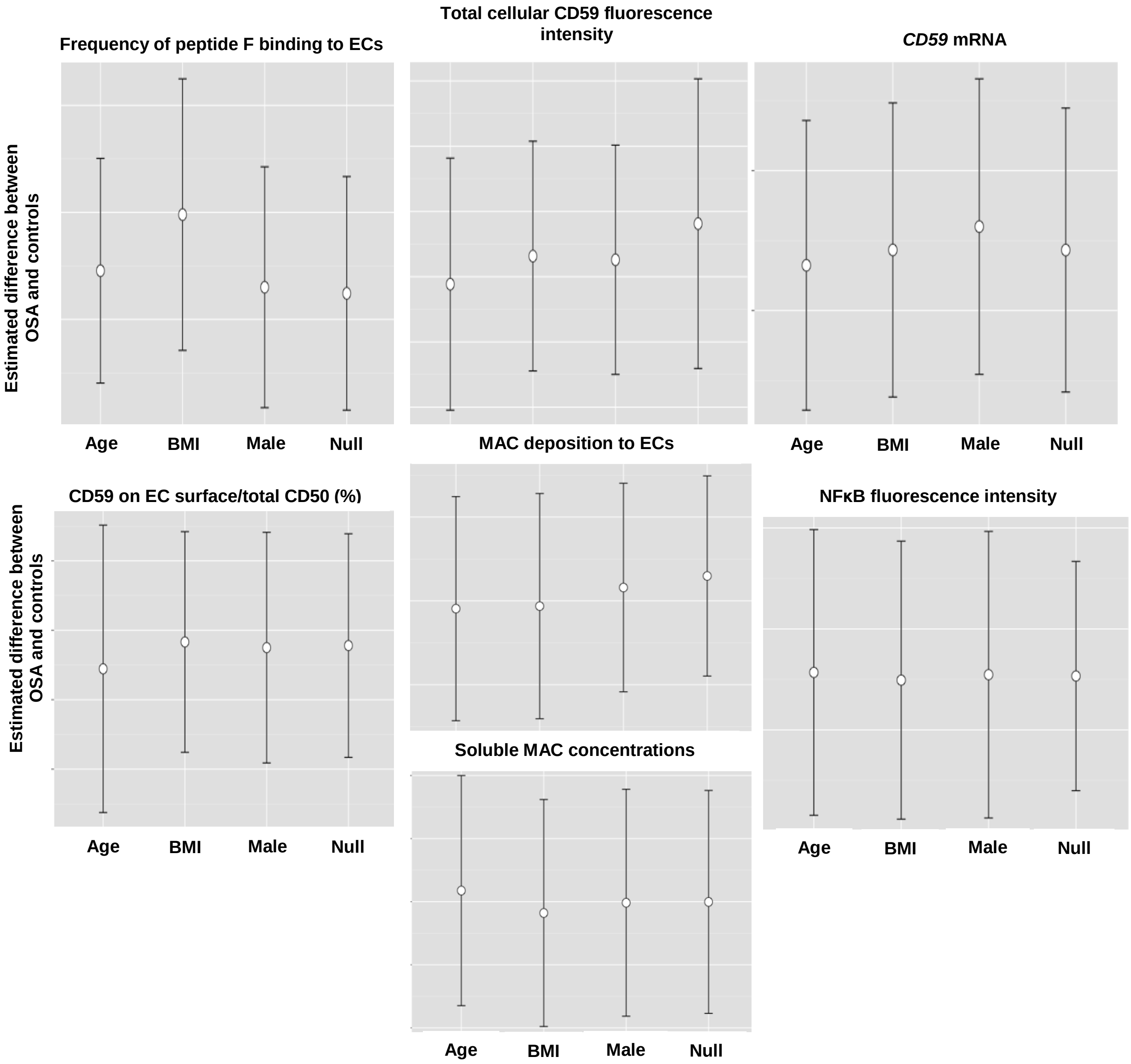

Fig. S2. Linear regression analysis. The graphs show estimated differences in measured variables between OSA patients and controls and their 95% confidence intervals from the following 4 models: null = model without adjustment for confounders; age = model adjusted for age; BMI = model adjusted for BMI; male = model adjusted for gender.

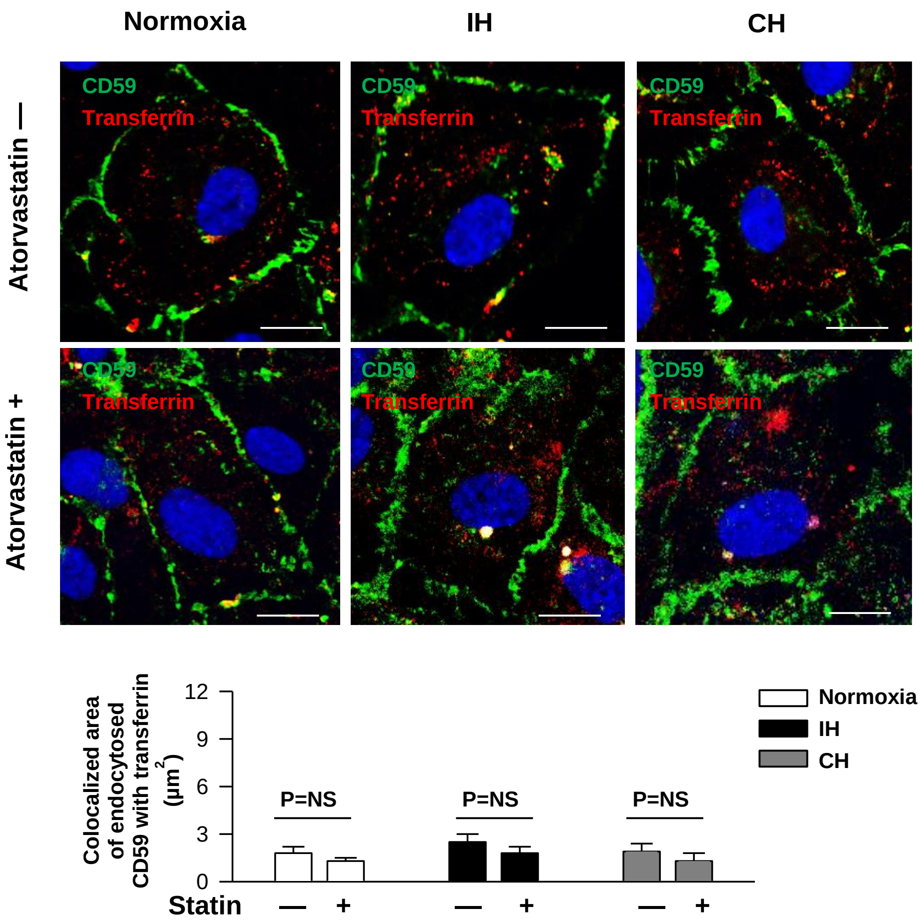

Fig S3. Endocytosis of CD59 with transferrin. Representative confocal images of CD59 endocytosis in HUVECs in normoxia, intermittent hypoxia (IH), or continuous hypoxia (CH) with or without atorvastatin (n=4). Scale bar 10 µm. Bar graph quantitates colocalized area of endocytosed CD59 with transferrin (μm^2). All data throughout the figure are shown as the mean ± SE, 2-sided Exact permutation test.

CH = Continuous Hypoxia; HUVEC = Human Umbilical Vein Endothelial Cells; IH = Intermittent Hypoxia. NS=not significant.

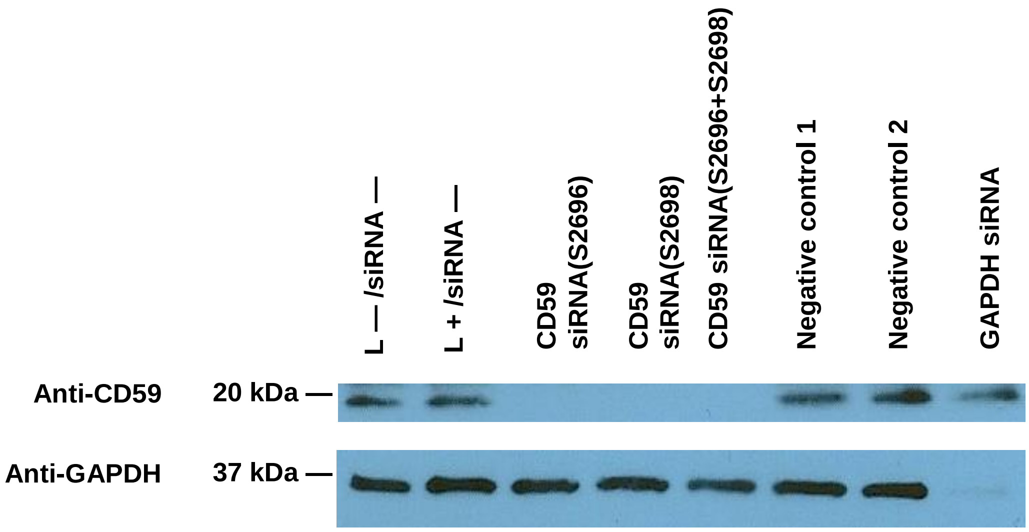

Fig S4. Confirmation of HUVEC transfection with CD59 siRNA. Western blots probed with antibodies directed against CD59 and glyceraldehyde 3-phosphate dehydrogenase (GAPDH). Transfection of HUVECs was carried out for 48 hours using lipofectamine (L) and siRNAs s2696, s2698. and a combination of s2696 and s2698 to knock down CD59. Two scrambled siRNAs served as a negative control, and siRNA against GAPDH served as a positive control (n=3). The expression of CD59 was almost completely knocked down by either siRNA (s2696 or s2698).

GAPDH = glyceraldehyde 3-phosphate dehydrogenase; HUVEC = Human Umbilical Vein Endothelial Cells.

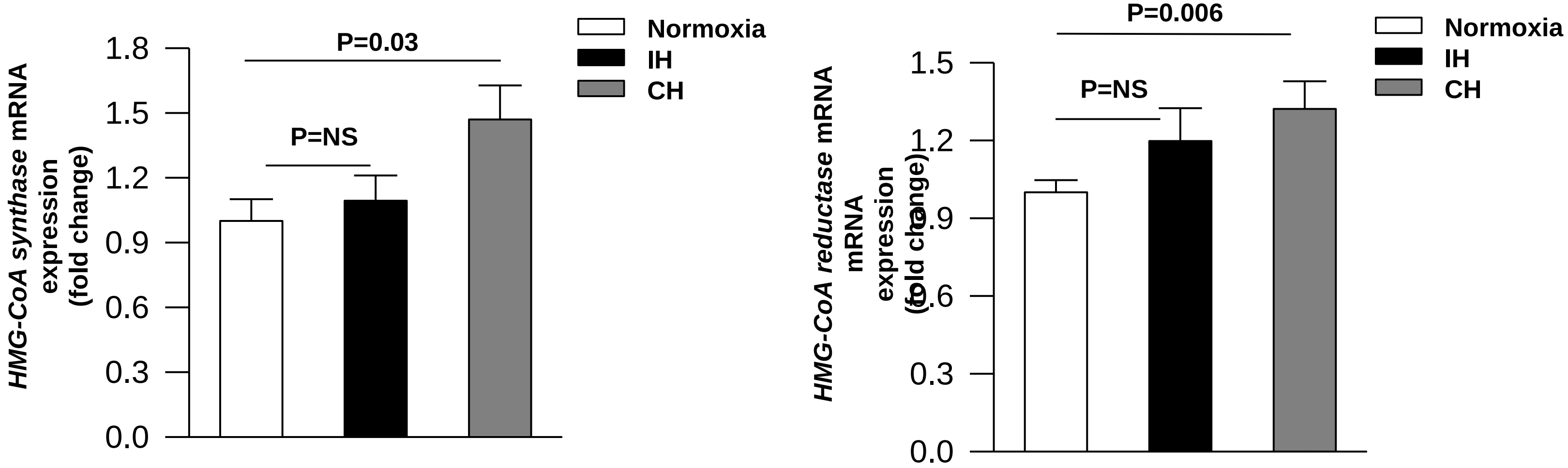

Fig S5. Cholesterol biosynthesis in IH. Quantitation of the *3-hydroxy-3-methylglutaryl-coenzyme A (HMG-CoA) reductase* and *synthase* mRNA expression in HUVECs in normoxia, intermittent hypoxia (IH), and continuous hypoxia (CH) expressed as fold change over normoxia (n=4). All data throughout the figure are shown as the mean ± SE, 2-sided Exact permutation test. CH = Continuous Hypoxia; HUVEC = Human Umbilical Vein Endothelial Cells; IH = Intermittent Hypoxia; NS=not significant.

Table. S1. Baseline characteristics of patients with OSA and control subjects.

	OSA patients (n=76)	Control subjects (n=52)	p value
**Age** (years)	45±1.6	39±1.6	0.01
**Body mass index** (kg/m^2)	37±1.0	34±1.4	NS
**Apnea-hypopnea index** (events/hour of sleep)	26±2.6	1.6±0.2	<0.001
**Oxygen Desaturation Index** (events/hour of sleep)	16±2.8	1.0±0.2	<0.001
**Epworth sleepiness scale score**	11±0.7	9±0.8	NS
**Systolic blood pressure** (mmHg)	121±2.1	123±2.3	NS
**Diastolic blood pressure** (mmHg)	77±1.3	77±1.5	NS

Data are presented as mean ± SE

	OSA patients (n=76)	Control subjects (n=52)	p value
**Gender** (% female)	50	51	NS
**SaO_2 nadir** (%)	85±0.8	91±0.8	<0.001
**t < SaO_2 90%** (% of the total sleep time) *	1.4±0.5	0±0.0	<0.001
**Hypertension, n** (%)	17 (22)	9 (17)	NS
**Dyslipidemia, n** (%)	10 (13)	5 (10)	NS

Data are presented as %.

SaO_2= arterial oxyhemoglobin saturation.

* Time spent below SaO_2 of 90% during sleep.

NS= not significant.

Table. S2. Phages isolated after panning without target cells and with bovine serum albumin in polystyrene wells.

**Negative template**
NWKPYPT
IVLPYPI
QFQHSHP
TPITQLL
IVLPYPI
THPPNAL
YLPLYEL
